# Exploring the Benefits of Doll Play Through Neuroscience

**DOI:** 10.3389/fnhum.2020.560176

**Published:** 2020-10-01

**Authors:** Salim Hashmi, Ross E. Vanderwert, Hope A. Price, Sarah A. Gerson

**Affiliations:** ^1^Department of Psychology, Institute of Psychiatry, Psychology and Neuroscience, King’s College London, London, United Kingdom; ^2^School of Psychology, Cardiff University, Cardiff, United Kingdom; ^3^School of Psychology, Cardiff University Centre for Human Developmental Science (CUCHDS), Cardiff, United Kingdom; ^4^School of Psychology, Cardiff University Brain Research Imaging Centre (CUBRIC), Cardiff, United Kingdom

**Keywords:** development, social processing, empathy, play, fNIRS (functional near infrared spectroscopy)

## Abstract

It has long been hypothesized that pretend play is beneficial to social and cognitive development. However, there is little evidence regarding the neural regions that are active while children engage in pretend play. We examined the activation of prefrontal and posterior superior temporal sulcus (pSTS) regions using near-infrared spectroscopy while 42 4- to 8-year-old children freely played with dolls or tablet games with a social partner or by themselves. Social play activated right prefrontal regions more than solo play. Children engaged the pSTS during solo doll play but not during solo tablet play, suggesting they were rehearsing social cognitive skills more with dolls. These findings suggest social play utilizes multiple neural regions and highlight how doll play can achieve similar patterns of activation, even when children play by themselves. Doll play may provide a unique opportunity for children to practice social interactions important for developing social-emotional skills, such as empathy.

## Introduction

Although children’s play is studied extensively, a definition as to what play is has not yet reached consensus (for a review, see Lillard, 2014). When children are asked, they describe playing as simply something they find fun (Downey et al., 2007). It is however generally agreed that play appears in many different forms including pretend or symbolic play, games with rules, language play, rough-and-tumble play, and construction play (Burghardt, 2010).

One of the more recognized and researched forms of play is “pretend play” (or symbolic play or fantasy play), where children playfully distort reality to behave in a nonliteral, “as if” mode (Fein, 1981). A common element of children’s pretend play is the presence of toys and dolls which act to encourage children’s pretense (Singer and Singer, 1990). Pretend play was originally argued to emerge when children reach the age of two and thereafter declines between the ages of four and seven (Piaget, 1962). However, it is increasingly recognized that play, and pretend-play in particular, continues beyond this age (e.g., Singer and Singer, 2005; Lillard, 2014).

Pretend play is argued to provide benefits in the development of social processing (Lillard, 2017) and executive function (see Carlson and White, 2013; Sachet and Mottweiler, 2013). Regular play with others provides advantages in aspects of social understanding, in terms of references to the thoughts and feelings of others (Youngblade and Dunn, 1995; Howe et al., 2014; Tessier et al., 2016), perspective taking (Dunn and Cutting, 1999; Harris, 2000), and empathy (Brown et al., 2017). However, the correlational nature of these studies limit conclusions regarding causation (see Lillard et al., 2013). In terms of children’s executive function, evidence from both correlational and intervention studies have found “pretend-play” to be associated with improvements in executive function skills (Albertson and Shore, 2009; Kelly and Hammond, 2011; Thibodeau et al., 2016), as children must inhibit reality to maintain the imagined components of play (Carlson et al., 2014), and use their working memory to retain and recall information regarding their play (Pierucci et al., 2014).

Although, play is considered a largely social activity (Lillard, 2017), pretend play can occur in both social contexts with a play partner and in the solitary form (Garvey, 1974), and solitary play is considered to be a preference for some children (Coplan et al., 2014; Ooi et al., 2018). Indeed, in one survey of children between the ages of 4 and 12, over a third of children reported playing with dolls and toys as one of their favorite activities, but only when playing alone, and this was mostly reported by the younger children (Downey et al., 2007). However, Piaget (1962) contended that all pretend play activities are social to an extent, as even solitary pretend play is a performance to an imaginary other.

### Play in the Brain

Limited research has investigated brain activation during children’s play. Due to the practical challenges of measuring brain activity during natural play, most research into the neural correlates of social interactions has used highly controlled tasks that have examined electrical activity (electroencephalography, EEG) or blood flow (functional near infrared spectroscopy, fNIRS) during episodes of brief social interaction or observation of social stimuli (e.g., mutual gaze or infant-directed speech) vs. lack of social interaction or observation of social stimuli (e.g., lack of eye contact or non-social stimuli). For example, Lloyd-Fox et al. (2009, 2015) have found activation in fNIRS optodes consistent with posterior superior temporal sulcus (pSTS) activation during processing of social and communicative stimuli, relative to non-social and non-communicative stimuli in infants and toddlers (Hakuno et al., 2018). These findings are consistent with pSTS being recruited during social interactions and social processing (e.g., the theory of mind) in functional magnetic resonance imaging (fMRI) studies in adults (Redcay et al., 2010; Lahnakoski et al., 2012; Deen et al., 2015). Whether naturalistic play activates these same brain regions is, as yet, unknown. Given that the pSTS is active during minimal social interactions in lab settings (e.g., shared attention on a toy), one would expect that active and natural play with another person would activate this region, especially when engaging in pretend play that enables social perspective-taking and representation of others’ emotions and thoughts.

The neural correlates of executive function are relatively well established across paradigms and ages. fMRI, EEG, and fNIRS research all indicate that the prefrontal cortex (PFC) is activated during executive functioning tasks that include inhibition and working memory (Burgess and Stuss, 2017). In preschool-aged children, for whom executive functioning skills are still emerging, individual differences in executive functioning correlate with differences in brain activation of this region. For example, in an fNIRS study with 3- and 5-year-olds, Moriguchi and Hiraki (2009) found that prefrontal areas were only activated during executive function tasks for those children who successfully performed the task and not those who made errors. Similar findings of individual differences in executive function skills relating to cortical activation of the PFC region have been found in adults using fNIRS (Yasumura et al., 2014). As far as we are aware, direct measurement of prefrontal activity during natural play that involves executive function skills like planning and inhibition has not been carried out.

The orbitofrontal cortex (OFC) is associated with reward processing and positive affect (Berridge and Kringelbach, 2008). For example, Minagawa-Kawai et al. (2009) found greater OFC activation (using fNIRS) when infants viewed their own mothers’ smiles than when they viewed an unfamiliar mother’s smile. In mothers, OFC activation was specific to viewing their infants, relative to unfamiliar infants, and was related to behavioral ratings of pleasant mood. Similar effects of rewarding and motivating stimuli on OFC have been found across multiple methodologies and ages (e.g., May et al., 2004; Kida and Shinohara, 2013). If children find certain kinds of play particularly rewarding and motivating, OFC activation should evidence this.

### Current Research

In the current research, we investigate the unique neural correlates of pretend play (in the form of doll play) relative to other play (tablet games) in a naturalistic setting. We collected fNIRS data from 4- to 8-year-old children while they engaged in varying forms of play either alone or with a social partner (i.e., an experimenter). Children in this age range were old enough to follow directions, play on their own, and maintain attention for the duration of the task but were young enough to engage in natural play in these settings. As a contrast to doll play, tablet games were chosen that allowed creative and open play (i.e., no set rules or objectives) and were suitable for this age range but did not involve doll play. In the chosen tablet games (described in more detail in the “Materials and Methods” section), children cut and styled hair or built towns. We included both solo and joint play to examine whether brain activity was different when children engaged in these forms of play by themselves or with a social partner. Our regions of interest (ROIs) covered elements of functional networks related to empathy and perspective taking (pSTS; Hakuno et al., 2018), executive function (PFC; Moriguchi and Hiraki, 2009), and reward-seeking (OFC; Minagawa-Kawai et al., 2009). Our key questions concerned which brain areas would be selectively engaged during doll play, relative to tablet play, and whether this was consistent across the solo and joint play.

## Materials and Methods

### Participants

Forty-two typically developing children aged between 4- and 8-years-old (*M* = 5.5 years, *SD* = 1.2; 22 females) were recruited *via* a participant database of volunteer families interested in participating in research in the local region. We were able to acquire full fNIRS data from 33 of the participants. Participants were excluded because of insufficient data (*n* = 5), experimenter or equipment failure resulting in bad fNIRS recordings (*n* = 3), or a statistical outlier in hemoglobin concentrations (>2 *SD* in multiple channels; *n* = 1). The excluded participants were noted to have touched the cap frequently or to have pulled sensors out during testing.

Participants were excluded from recruitment if their parents had described them as having neurological abnormalities, developmental delays, or special education needs. Written informed consent was obtained from the participant’s parent or caregiver before the start of the experiment. Participants were given a certificate and a prize worth approximately £10 for their participation. The ethical review panel in the School of Psychology at Cardiff University reviewed and approved all procedures and written informed consent to participate in this study was provided by the participants’ legal guardian (approval: EC.19.06.11.5641RA).

### Procedure

Before the start of the experiment, children were given the chance to familiarize themselves with an experimenter by playing with them in a reception area. The child’s head was then measured to ensure that the correct cap size was set-up. The parent and child were then guided to the testing room where the child was asked to sit on a carpet square on the floor and to face a wall-mounted computer monitor.

Once seated, children were capped and the lighting in the room was dimmed to allow for better data acquisition. While the primary experimenter carried out capping and calibration of the fNIRS equipment, the familiar experimenter briefly showed the child how to play the two tablet games to ensure that children knew how to play the games without assistance. Once the primary experimenter had achieved a good signal quality, the parent or caregiver was encouraged to observe from an adjoining room. If a parent preferred to stay in the room, they were prompted not to interact with their child, and to sit on a chair in the corner of the room; we confirmed *via* a video recording that parents did not interfere with the task.

The fNIRS testing session began with the child sitting quietly and watching a 5-min space video. Then the play blocks began. In the first two blocks, the child and the familiar experimenter played together with dolls and with the tablet. The child then played by themselves for the next six blocks, alternating between doll-play and tablet-play. The session concluded with two blocks while the child and familiar experimenter played together with the dolls and tablet. The order of presentation of tablet games and doll sets was counterbalanced barring the last two blocks where the child got to choose their favorites to play with a second time.

Children were allowed to take breaks during testing or could stop the session early if desired. The entire testing session lasted approximately 60 min.

### Materials

#### Parent Questionnaire

While children were engaged in the task, parents were asked to complete a short questionnaire regarding their child’s experience with tablets and dolls. Parents reported whether or not their children played with tablet devices and dolls at home and school/daycare. They were asked open questions about how often their child played with the toys, whether they play independently or socially, and what forms of toys (tablet games and types of dolls) children played with at home. They were asked to rate how much their child enjoyed each type of play on a Likert scale from 1 (not at all) to 5 (very much).

#### Task and Stimuli

**Figure 1:**
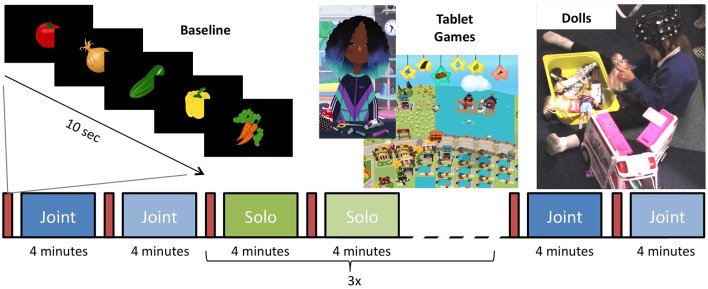
Experimental paradigm for the testing session. Between every play block, there was a 10-s baseline of vegetables that flashed on the screen. The session began with one joint play block of tablet and doll play. Three blocks of solo tablet and three blocks of solo doll play alternated before the session ended with one joint tablet and one joint doll play blocks.

Timing and order of the play blocks were controlled by E-Prime 3.0 (Psychological Software Tools, Sharpsburg, PA, USA) and stimuli were presented on an IIyama ProLite 24 "LCD monitor. The primary experimenter controlled the beginning of each block with a button press once the child was ready to begin. During each play session, either doll or tablet, the screen was black with small text in the corner indicating the current block and what the next block would be; to prepare and minimize transition time between blocks. Before each play block, a 10-s baseline video of five pseudorandom images of clipart vegetables (broccoli, onions, carrots, pumpkin, eggplant, radishes, and cucumber) on a black background was presented on the center of the screen for 1.5 s each, interspersed with a white fixation cross on for 0.5 s (Figure 1). Following the baseline video, the materials for the play block were then set up in the room.

#### Joint Play

At the beginning and end of the session, participants engaged in joint play sessions with the experimenter. Each play session lasted 4 min and the order in which the child engaged in doll vs. tablet play session was counterbalanced between participants. The first doll block was always the family set. Following the solo play blocks, the child was prompted to again play with the experimenter. For these blocks, the child chose their favorite set of dolls and tablet games to play with them again. During all joint play sessions, the second experimenter allowed the child to lead the play session.

#### Solo Play

The joint play sessions were followed by six solo play sessions, each lasting 4 min. The sessions alternate between doll and tablet play and the order in which the different doll sets and the two tablet games were presented was randomly determined. During solo play sessions, the secondary experimenter sat behind the participant and did not engage with them. A similar method has been used in previous research and has been found not to influence children’s play (Krafft and Berk, 1998). If participants attempted to engage them, they responded as briefly as possible.

#### Tablet Games

Toca Hair Salon 3 (Toca Boca, Stockholm, Sweden) and Hoopa City 2 (Dr. Panda, Chengdu, China) were selected because they were engaging, open-ended, and did not involve any stringent rules. These criteria were chosen to align tablet-play style with doll-play on creativity and child guided play. Toca Hair Salon 3 is a hairdressing game in which players can wash, cut, and style one of four character’s hair. Hoopa City 2 is a city-building game in which players can place roads, buildings, and parks onto a map (Figure 1). The tablet games were played on a 12-inch iPad 3 IOS 9.3.5.

#### Doll Sets

Four different sets of dolls were used in the current experiment: the family set, the careers set, the estate set, and the animals set (see Supplementary Materials). The sets were made up of several Barbie (Mattel Co., El Segundo, CA, USA) playsets and additional individual dolls. Before each participant, the dolls were checked and returned to their starting positions to ensure consistency across participants.

### Video Recording

The experiment was recorded using both a Logitech C270 720p Webcam attached to the monitor and a Canon LEGRIA HF R706 camera mounted on a tripod in the corner of the room to view play over the child’s shoulder. This allowed the capture of both the child’s facial expressions and actions during play. The cameras were adjusted before the start of each experiment to ensure that the child was captured.

### fNIRS Data Acquisition

The measurement of the concentration changes in oxygenated hemoglobin (oxy-Hb) and deoxygenated hemoglobin (deoxy-Hb) was carried out *via* the NIRScout fNIRS system and NIRStar software (NIRX, Medizintechnik, GmbH, Berlin, Germany). The system operated at both 760 and 850 nm wavelengths, with a scan rate of 3.91 Hz. Sixteen sources and 16 detectors were used for this experiment, making a total set of 41 source-detector pairs. The sources and detectors were inserted into a flexible nylon NIRScap (NIRX) worn by the participant for the duration of the experiment. The distance between the sources and detectors was fixed at 3 cm.

**Figure 2:**
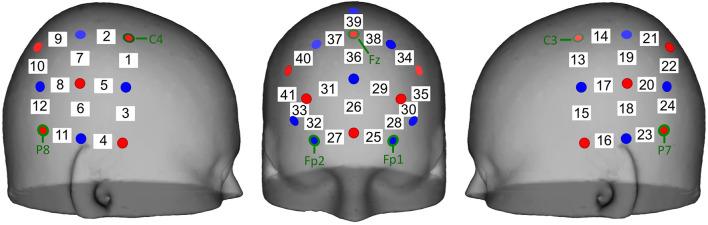
Schematic of optode placement on the scalp and the estimated channel locations used for data analysis. Bilateral panels of five sources (red) and four detectors (blue) separated by 3 cm were placed centered on the international 10-20 system sites CP5 and CP6 resulting in 12 channels (white) per hemisphere. A frontal panel of six sources and eight detectors separated by 3 cm was placed with the bottom row of optodes over the Fp1, Fpz, and Fp2 sites.

The positioning of the source-detector pairs was customized for this experiment, with the optode pairs covering the frontal, temporal, and parietal cortices (Figure 2). The optodes were positioned in such a way to record oxy-Hb and deoxy-Hb concentrations in the following ROIs: orbitofrontal (OFC) and prefrontal (PFC) cortices in the front and bilateral pSTS in the left and right hemispheres. The cap was placed so that the front seam rested just above the participant’s eyebrows and the participant’s ears pulled through the earholes on both sides maintaining a consistent cap placement.

### fNIRS Data Processing

Processing of the fNIRS data was carried out *via* nirsLAB v.2019.04 (NIRX, Medizintechnik, GmbH, Berlin, Germany) following the reporting recommendations by Pinti et al. (2019). Brief spikes or discontinuities (i.e., <1 s in duration) in the raw optical time-series data were manually identified and interpolated in all channels. Each channel with a gain setting greater than 6 (maximum system gain = 7) was then visually inspected and channels with excessive noise were removed from further analysis. A finite impulse response bandpass filter from 0.03 to 0.8 Hz was then applied to the optical data with a 15% roll-off. These filter cutoffs were based on previous research with similar designs (Gervain et al., 2008; Perdue et al., 2014; Ravicz et al., 2015) and were aimed to remove slow drift and higher heart rate fluctuations. The optical data were then converted into hemodynamic states using the modified Beer–Lambert Law.

Hemodynamic data were baseline corrected to the preceding 20-s before the onset of each play block (including baseline and set up for subsequent block). Mean Oxy-Hb and deoxyHb concentrations were first averaged across similar blocks of joint and solo doll or tablet play, then the concentrations were averaged across the 4-min blocks. Finally, because we did not measure the precise placement of the optodes, we averaged the activity in clusters of optodes overlying our ROI: left OFC (25, 28, 30), right OFC (27, 32, 33), left PFC (34, 35), right PFC (40, 41), left pSTS (14, 18, 19, 20, 21, 22), and right pSTS (2, 6, 7, 8, 9, 10). These regions were defined based on previous fNIRS research and research that co-registered MRIs with fNIRS to identify underlying regions (Lloyd-Fox et al., 2009, 2014).

### Statistical Analysis

To examine the differences in neural activation during joint or solo play with dolls or tablet games, we conducted separate analyses using a within-subjects 2 Social Context (Joint/Solo) × 2 Play Type (Doll/Tablet) × 2 Hemisphere (Left/Right) repeated measures ANOVA (rmANOVA). We measured activation in anatomical fields covering elements of functional networks related to empathy and perspective taking (pSTS), executive function (PFC), and reward-seeking (OFC). For all tests, *α* < 0.05 was considered significant. We also included child sex as a between-subjects factor in all models to explore whether brain activation is consistent between boys and girls. In preliminary analyses, we also included age as a covariate. As no main effects or interactions with age emerged, we removed this variable from subsequent analyses because it reduced power. All significant interactions were examined using *post hoc* *t*-tests.

Statistical tests were conducted for both oxy-Hb and deoxy-Hb and results from the deoxy-Hb can be found in Supplementary Materials.

## Results

### Questionnaire Responses

Thirty (90.91%) of the 33 children had parents complete the questionnaire on their experience with tablets and dolls. Of these, 29 (96.67%) parents reported that their children used a tablet at home and 22 (73.33%) reported that their child played with dolls at home. Twenty-two (73.33%) parents reported that their children used a tablet at school [two (6.67%) reported they did not; six (20%) reported that they did not know], and 12 (40%) reported that they played with dolls at school [seven (23.33%) reported they did not; 11 (36.67%) reported that they did not know]. The one parent who reported that their child did not play with a tablet device at home reported that their child did use a tablet at school. Based on this evidence, we were confident that children would be proficient in using a tablet during both solo and joint play and that a lack of familiarity with either type of toy would not drive any differences between conditions.

### Posterior Superior Temporal Sulcus

**Figure 3:**
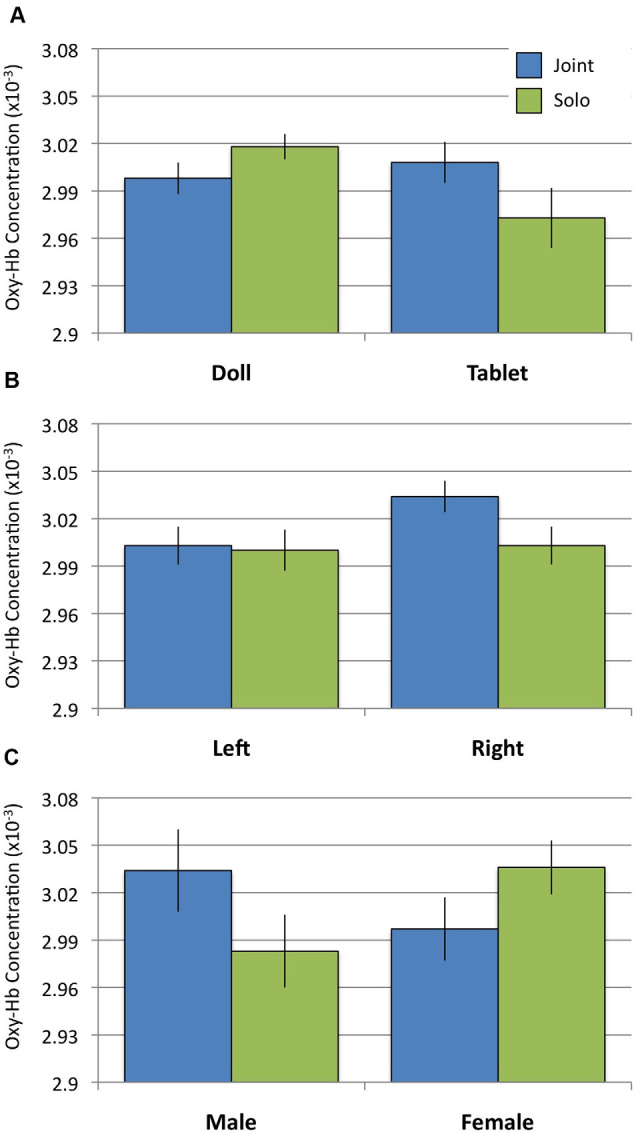
Oxygenated hemoglobin (Oxy-Hb) concentrations during Joint and Solo play. **(A)** Activation in the posterior superior temporal sulcus (pSTS) for doll and tablet blocks. **(B)** Activation in prefrontal cortex (PFC) in the left and right hemispheres. **(C)** Activation in the orbitofrontal cortex (OFC) between males and females.

**Figure 4:**
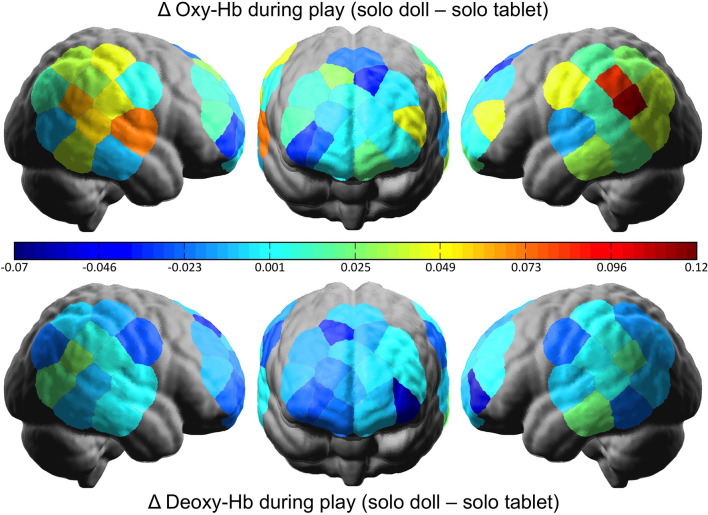
The difference in cortical activation between doll and tablet play in the solo social context. Greater values represent more oxygenated (above) and deoxygenated (below) hemoglobin in the doll relative to the tablet play.

To examine whether or not doll play activates more social regions of the brain relative to tablet play, we conducted a 2 Social Context × 2 Play Type × 2 Hemisphere rmANOVA using oxy-Hb concentrations from the pSTS regions. Results revealed a significant interaction between Social Context and Play Type (*F*_(1,31)_ = 5.242, *p* = 0.029, $\eta_{\text{p}}^{2}$ = 0.145). *Post hoc* analyses revealed no differences in pSTS activation during joint play (doll: *M* = 3.00, *SD* = 0.057; tablet: *M* = 3.01, *SD* = 0.074; *t*_(31)_ = 0.667, n.s.). During solo play, dolls (*M* = 3.02, *SD* = 0.045) elicited significantly greater activation than tablet play (*M* = 2.97, *SD* = 0.107; *t*_(31)_ = 2.368, *p* = 0.024; Figures 3A, 4). There were no significant effects or interactions involving the child’s sex.

### Prefrontal Cortex

To examine whether or not tablet play activates regions of the brain associated with behavioral control (i.e., executive function), we conducted a 2 Social Context × 2 Play Type × 2 Hemisphere rmANOVA using oxy-Hb concentrations from the PFC region. Results revealed a significant main effect of Hemisphere (*F*_(1,31)_ = 5.995, *p* = 0.020, $\eta_{\text{p}}^{2}$ = 0.162) qualified by a significant Social Context × Hemisphere interaction (*F*_(1,31)_ = 6.161, *p* = 0.019, $\eta_{\text{p}}^{2}$ = 0.166). *Post hoc* analyses of the interaction revealed significantly greater activation in the right PFC during joint play (*M* = 3.034, *SD* = 0.057) relative to solo play (*M* = 3.003, *SD* = 0.068; *t*_(31)_ = 2.583, *p* = 0.015) and relative to activity in the left PFC (joint: *M* = 3.003, *SD* = 0.068; *t*_(31)_ = 3.00, *p* = 0.005; solo: *M* = 3.000, *SD* = 0.074; Figure 3B) across both doll and tablet play. There were no significant effects or interactions involving the child’s sex.

### Orbitofrontal Cortex

To examine whether doll or tablet play activates regions of the brain associated with processing rewarding events, we conducted a 2 Social Context × 2 Play Type × 2 Hemisphere rmANOVA using oxy-Hb concentrations from the OFC region. Results revealed a significant Social Context × Sex interaction (*F*_(1,31)_ = 4.283, *p* = 0.047, $\eta_{\text{p}}^{2}$ = 0.121). *Post hoc* analyses of the interaction suggest females (*M* = 3.036, *SD* = 0.096) had greater OFC activation relative to boys (*M* = 2.983, *SD* = 0.130) during solo play, however, the contrast was not significant (*t*_(31)_ = 1.893, *p* = 0.068). There were no differences between girls (*M* = 2.997, *SD* = 0.113) and boys (*M* = 3.034, *SD* = 0.147) in OFC during joint play (*t*_(31)_ = 1.121, n.s.) or between joint and solo play in either group (Figure 3C).

## Discussion

This is the first experiment to directly test the neural correlates of play in young children. We found that the pSTS, a brain region associated with social processing and empathy, is activated when children play with a social partner, regardless of whether that play is with dolls or a tablet. Interestingly, however, when playing alone, this region is more engaged during doll play than tablet play. This supports behavioral findings that pretend play supports social processing and empathic reasoning (Dunn and Cutting, 1999; Brown et al., 2017) and raises new queries regarding the benefits of solo vs. social play.

That pSTS activity did not differ between play forms when children played with a social partner suggests that children can rehearse social perspective-taking and empathy when playing with a partner, regardless of whether that play takes the form of pretend play with dolls or creative play on a tablet. This is consistent with findings suggesting that screen-time is most beneficial for social and cognitive development when carried out interactively (e.g., Supanitayanon et al., 2020).

The interaction between social context and play type was driven by the fact that, when playing alone, there was more pSTS activity for a doll than tablet play. This provides support for Piaget’s (1962) classic claim that all pretend play is inherently social in that it allows the rehearsal of social interactions and social perspective taking (Harris, 2000). Pretend play with dolls therefore provides a unique outlet for practicing social and empathic skills even when playing by oneself.

There were no differences in terms of PFC activation, associated with executive functioning, between doll and tablet play. This implies that children did not recruit executive function skills differentially when playing with different toys. Although these findings contrast previous research finding associations between executive function and pretend play in preschool-aged children (e.g., Albertson and Shore, 2009; Kelly and Hammond, 2011), they are in line with research findings in older children where these associations are not found (Hoffman and Russ, 2012). An interaction between the hemisphere and social context of play indicated that the right PFC was more activated during joint than solo play (but this was not the case for left PFC). This suggests that social play requires more behavioral control than solo play, but why this effect is specific to the right hemisphere is an open question.

In terms of reward processing, no difference in OFC activation was found between different forms of play, but a gender by social context interaction indicated that there was marginally less activation for boys than girls during solo play. This implies that boys might find solo play less rewarding than joint play, whereas girls find solo and joint play equally rewarding. This should be interpreted with caution, however, given that the *post hoc* tests were only marginally significant.

The findings from this experiment are unique in that they measured brain activity during live, natural play. The play was open-ended and no instructions were given to children except to play how they would like. The fact that the pSTS, a social processing region, was activated during open-ended play thus bolsters previous laboratory-based findings indicating that this region is important for social interactions, social processing, and empathy (Lloyd-Fox et al., 2009, 2015; Redcay et al., 2010; Lahnakoski et al., 2012; Deen et al., 2015; Hakuno et al., 2018). Doll and tablet play sessions were designed such that both would allow free, creative play with no set goals or objectives. Although doll play is often categorized as an activity for girls rather than boys, we found no gender differences in brain activity when playing with either dolls or tablets. This suggests that the benefits of play are not unique to either gender.

These findings have implications for potential interventions. Previous research in 4- to 7-year-old children has found that a preference for playing alone in various play activities is associated with teachers’ ratings of the children’s behavior as asocial, experiencing peer exclusion, and is negatively associated with mother’s ratings of their social engagement (Coplan et al., 2014; Ooi et al., 2018). Whilst it could be that children prefer to play alone because they experience peer exclusion, it could also be that those who prefer solitary play do not gain the advantage in social skills afforded by social play. If pretend play with dolls does help children practice these social skills without the threat of exclusion or rejection, this could be one avenue to improve social functioning in these children.

Although measuring brain activity during natural play has many advantages, it also limits the conclusions we can draw from the current findings. Whether particular brain activity reflects rehearsal of the skills typically associated with that region cannot be directly assessed in the current experiment. Future research should build on the current findings by assessing whether individual differences in brain activity related to variability in behavior that reflects practicing these skills (e.g., empathy, perspective-taking, and executive function), and whether there is a subsequent improvement in these skills.

This research provides the first evidence that social processing brain regions are similarly active during pretend play with dolls both when playing alone or with a social partner. The fact that pSTS activation is stronger for doll play than tablet play specifically when playing alone is consistent with the notion that pretend play allows children to practice social interactions even when playing by themselves. The implications of these findings for those interested in play, neuroscience, and social development are far-reaching and are suggestive that research investigating the short- and long-term consequences of pretend play on both brain and behavior will be fruitful.

## Data Availability Statement

The raw data supporting the conclusions of this article will be made available by the authors, without undue reservation.

## Ethics Statement

The studies involving human participants were reviewed and approved by Ethical Review Panel, Cardiff University School of Psychology. Written informed consent to participate in this study was provided by the participants’ legal guardian/next of kin.

## Author Contributions

SH, RV, and SG designed the study. RV, HP, and SG conducted the experiment. RV and HP processed the data. RV conducted the analyses. All authors contributed to the article and approved the submitted version.

## Conflict of Interest

Funding for this project was provided by Mattel Inc^®^ through partnership with Oxy Insight Ltd. Mattel and Oxy Insight funded the equipment and some personnel for this project and were involved in initial discussions regarding the study design. Neither Mattel or Oxy Insight had final say in the study design nor played a direct role in the collection or analysis of the data; interpretation of the results; or writing of the manuscript.
