# Supplementary material for: Exploring the Benefits of Doll Play Through Neuroscience
Source: Front Hum Neurosci. 2020 Oct 1;14:560176. doi: 10.3389/fnhum.2020.560176 (PMC7560494; doi:10.3389/fnhum.2020.560176)
Supplement: Supplementary file 1 [file Image_1.PDF]

Supplemental Methods

Doll sets. Supplemental Table 1 is the description of the specific sets of dolls and accessories used for each block of testing. Supplemental Figure 1 displays photos of those sets of dolls and accessories for each block of testing. The *Family Set* was always used for the first Joint Doll play block and the order of the *Careers*, *Animal*, and *Estate Sets* for the Solo Doll play was counterbalanced across children. The child was able to choose their preferred set for the final Joint Doll play block.

Supplementary Table 1

Description of the Sets of Dolls and Accessories Used for Each Block of Testing

Careers Set	Animal Set	Estate Set	Family Set
-Barbie Care Clinic Playset<br><br>-Barbie Dreamtopia Sparkle Lights Mermaid<br><br>-Barbie Doctor Doll x2 (swapped one set of clothes with Fasionista 105)<br><br>-Barbie Fashionistas Doll 106<br><br>-Barbie Fashionistas Doll 105<br><br>-Ken Fashionistas Doll 118<br><br>-Barbie Astronaut Doll x 2	-Hugs 'n' Horses Chelsea & Barbie Playset<br><br>-Barbie Doggy Daycare Dolls & Pets<br><br>- Ken Fashionistas Doll 116	-Malibu House Playset with Accessories<br><br>-Barbie Skipper Babysitters Doll and Stroller Playset<br><br>-Barbie Fashionistas Doll 111<br><br>-Barbie Fashionistas Doll 113<br><br>-Barbie Fashionistas Doll 105<br><br>-Barbie Fashionistas Doll 110<br><br>-Ken Fashionistas Doll 14	-Barbie Skipper Babysitters Doll and Stroller Playset<br><br>-Playset with Bathtub, Babysitting Skipper Small Toddler Doll (adult doll from this set was swapped with an astronaut from the Careers Set)<br><br>- Ken Fashionistas Doll 114

Supplementary Figure 1

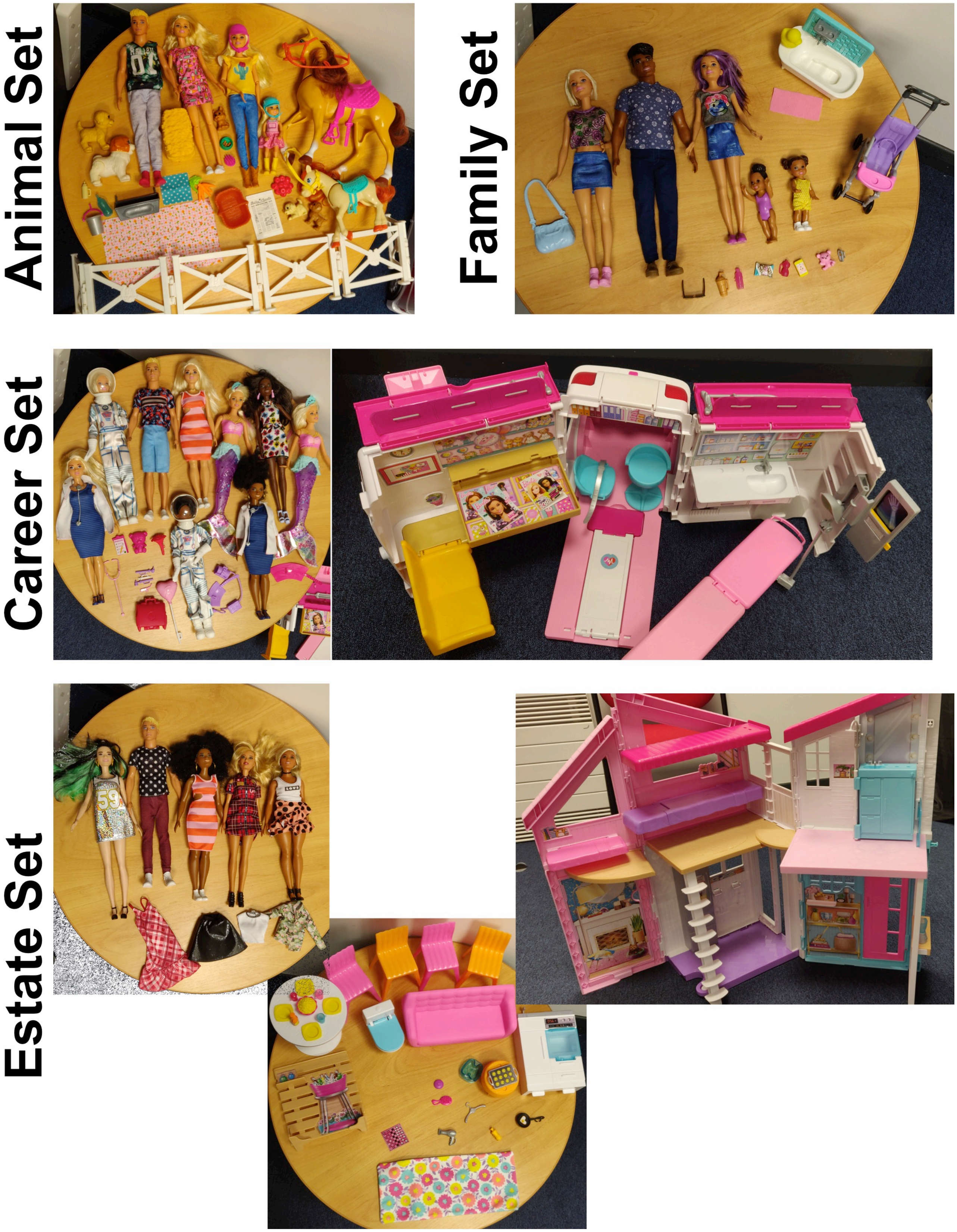

Supplemental Results (Deoxygenated Hemoglobin)

Posterior Superior Temporal Sulcus

To examine whether or not doll play activates more social regions of the brain relative to tablet play, we conducted a 2 Social Context x 2 Play Type x 2 Hemisphere rmANOVA using deoxy-Hb concentrations from the pSTS regions. There were no significant main effects or interactions apart from a trend significance Hemisphere x Social Context x Sex interaction ($F(1, 31) = 3.186$, $p = .084$, $\eta_p^2 = .093$).

Prefrontal Cortex

To examine whether or not tablet play activates regions of the brain associated with behavioral control (i.e., executive function), we conducted a 2 Social Context x 2 Play Type x 2 Hemisphere rmANOVA using deoxy-Hb concentrations from the PFC region. Results revealed a significant 3-way interaction between Social Context, Play Type, and Sex ($F(1, 31) = 11.015$, $p = .002$, $\eta_p^2 = .262$). Post hoc analyses of the interaction revealed that during solo doll play, Girls had significantly greater deoxy-Hb concentrations ($M = 3.018$, $SD = 2.40$) relative to Boys ($M = 2.997$, $SD = 1.152$; $t(31) = 2.10$, $p = 0.047$) in the PFC. Boys also had greater deoxy-Hb concentrations during joint doll play ($M = 3.019$, $SD = 1.008$) relative to joint doll play ($M = 2.997$, $SD = 1.152$; $t(31) = 2.20$, $p = 0.036$) in the PFC. Finally, Boys had greater deoxy-Hb concentrations during solo tablet play ($M = 3.027$, $SD = 0.864$) relative to solo doll play ($M = 2.997$, $SD = 1.152$; $t(31) = 3.222$, $p = 0.003$) in the PFC. All other contrasts were non-significant. There were also trend significant main effect of Play Type ($F(1, 31) = 3.569$, $p = .068$, $\eta_p^2 = .103$) and 3-way interaction between Social Context, Play Type and Hemisphere

($F(1, 31) = 2.948$, $p = .096$, $\eta_p^2 = .087$). All other main effects and interactions were non-significant.

Orbitofrontal Cortex

To examine whether doll or tablet play activates regions of the brain associated with processing rewarding events, we conducted a 2 Social Context x 2 Play Type x 2 Hemisphere rmANOVA using deoxy-Hb concentrations from the OFC region. Results revealed significant main effects of Social Context ($F(1, 31) = 8.793$, $p = .006$, $\eta_p^2 = .221$) and Play Type ($F(1, 31) = 5.493$, $p = .026$, $\eta_p^2 = .111$). Examination of the means for Social Context revealed greater deoxy-Hb during joint play ($M = 3.035$, $SD = 7.688$) relative to solo play ($M = 3.012$, $SD = 4.805$) in the OFC. Similarly, for the Play Type main effect, there was greater deoxy-Hb for tablet play ($M = 3.035$, $SD = 7.688$) relative to doll play ($M = 3.013$, $SD = 5.766$) in OFC. There were also trend significant 4-way interaction between Social Context, Play Type, Hemisphere and sex ($F(1, 31) = 3.478$, $p = .072$, $\eta_p^2 = .101$). All other main effects and interactions were non-significant.
